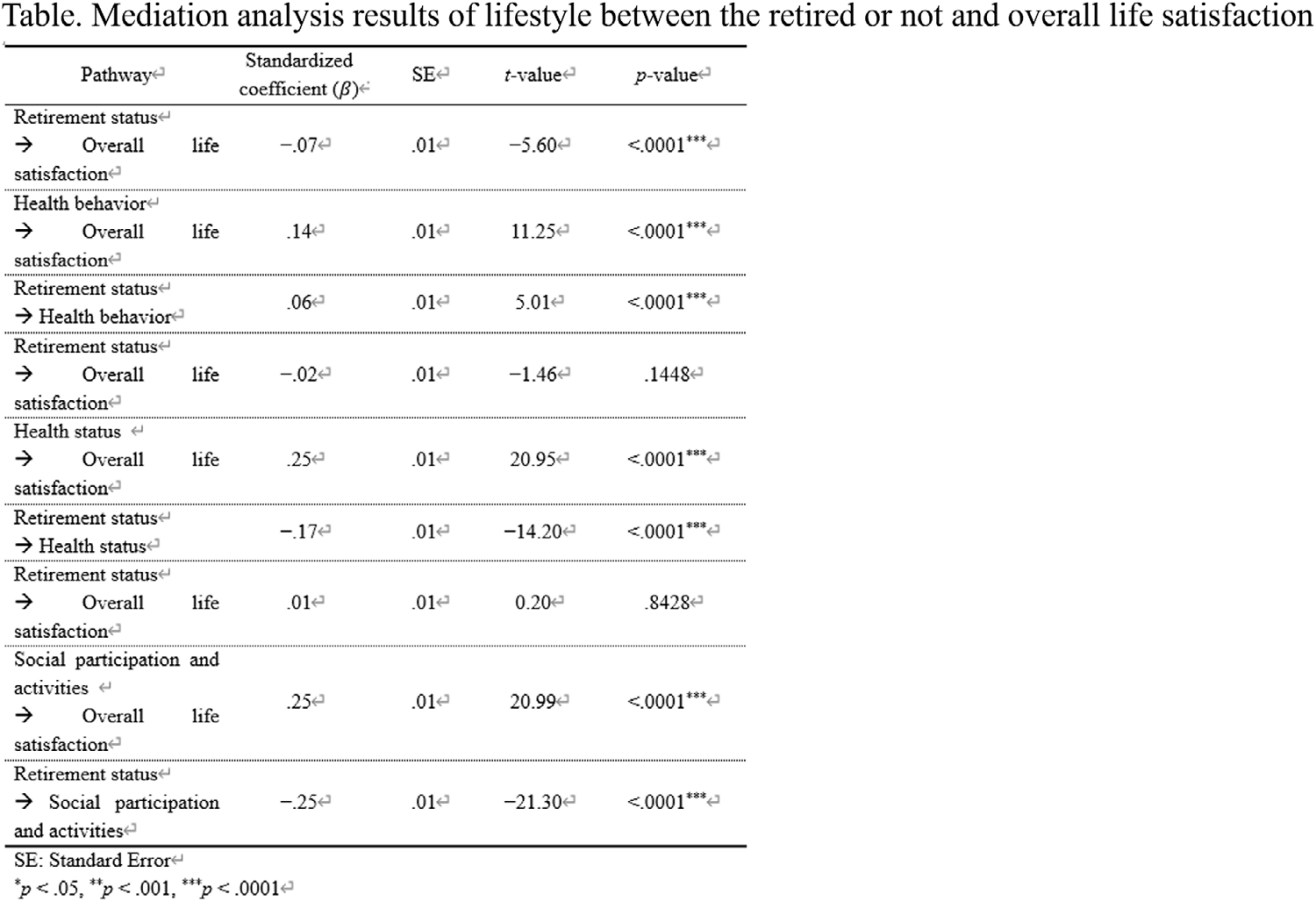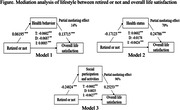# Retirement and Healthy Lifestyle: Mediating Effects on Life Satisfaction

**DOI:** 10.1002/alz.095419

**Published:** 2025-01-09

**Authors:** Sohyeon Yun, Hae Yean Park, Inhye Kim, Hyunseo An, Jiwon Shin, Hyun Yang, Sanghun Nam

**Affiliations:** ^1^ Graduate School, Yonsei University, Wonju, Wonju Korea, Republic of (South); ^2^ Yonsei University, Wonju Korea, Republic of (South); ^3^ Yonsei New‐normal Lfiestyle Resarch Center, Yonsei University, Wonju, Gangwon‐do Korea, Republic of (South)

## Abstract

**Background:**

Retirement for elderly people marks a critical turning point in life, acting as a significant personal and social event associated with both positive and negative health impacts. Studies on elderly populations from the successful aging perspective suggest that individual efforts and external support are necessary for successful aging. The WHO emphasizes the importance of a healthy lifestyle in achieving health and well‐being in human life and indicates the need for professional programs.

**Method:**

Data from 6,488 Korean adults, aged between 57 and 102 years, collected in the 2020 Korean data from the Korean Longitudinal Study on Aging (KLoSA), was used to analyze both direct and indirect effects of retirement status on life satisfaction. From the processed data, independent (retirement status), dependent (overall life satisfaction), mediating (lifestyle), and demographic characteristic variables were extracted for the mediation effect analysis. This process utilized the method of the mediating effect verification based on path analysis suggested by Baron and Kenny. To examine the statistical significance of the mediating role of lifestyle, a Sobel test was performed.

**Result:**

Health behaviors accounted for a 14% partial mediation, while health status and social participation and activities demonstrated 70% and 96% full mediation effects, respectively. Additionally, the Sobel test results confirmed that the mediating effects of all subdimensions of lifestyle in the relationship between retirement status and overall life satisfaction were significant (p < .0001).

**Conclusion:**

These results indicate that factors determining life satisfaction and successful aging at an individual level are significantly influenced not only by physical and psychological health but also by social competence, forming social relationships, and pursuing collective values, aligning with that of previous studies. To maintain optimal health status and behaviors, efforts to maintain health are required, namely, practicing successful aging. These findings imply the need for social prescription lifestyle programs to prepare for successful aging before retirement. A healthy lifestyle can be formed through various behavioral patterns and is shaped over the long term based on individualized contexts rather than showing short‐term changes. Therefore, a strategic approach focusing on both long‐term habit formation and new habits is necessary.